# Cartilaginous defect of the lateral trochlea following suprapatellar nailing of an open tibial shaft fracture: a case report

**DOI:** 10.1093/jscr/rjac144

**Published:** 2022-04-12

**Authors:** Henry A Kuechly, Cameron G Thomson, Ramsey S Sabbagh, Nihar S Shah, Jorge H Figueras, Brian M Grawe

**Affiliations:** Department of Orthopaedic Surgery and Sports Medicine, University of Cincinnati College of Medicine, Cincinnati, OH, USA; Department of Orthopaedic Surgery and Sports Medicine, University of Cincinnati College of Medicine, Cincinnati, OH, USA; Department of Orthopaedic Surgery and Sports Medicine, University of Cincinnati College of Medicine, Cincinnati, OH, USA; Department of Orthopaedic Surgery and Sports Medicine, University of Cincinnati College of Medicine, Cincinnati, OH, USA; Department of Orthopaedic Surgery and Sports Medicine, University of Cincinnati College of Medicine, Cincinnati, OH, USA; Department of Orthopaedic Surgery and Sports Medicine, University of Cincinnati College of Medicine, Cincinnati, OH, USA

## Abstract

A 34-year-old female sustained a 1.8 cm full-thickness chondral defect of the right lateral trochlear surface as the result of intramedullary tibial nailing via a suprapatellar portal to treat a displaced right sided open comminuted spiral fracture of the distal tibial shaft. An osteochondral allograft was used to treat the chondral defect. Iatrogenic injury to intraarticular structures is a potential complication when inserting a tibial nail via a suprapatellar portal. Using proper technique with cannula systems and guide pins is essential to lowering the risk of damage to intraarticular structures.

## INTRODUCTION

Intramedullary nailing is the gold standard for treatment of diaphyseal tibia fractures. The semiextended, suprapatellar technique has recently garnered favor over the traditional hyperflexed, infrapatellar approach for several well-deserved reasons [1, 2]. In addition to operative ease, the semiextended position mitigates the anterior pull of the proximal fracture fragment by the quadriceps tendon, leading to lower rates of malunion compared to the infrapatellar technique [3, 4]. Suprapatellar tibial nailing has also been shown to result in lower pain scores and higher functional outcomes compared to the infrapatellar approach [5, 6].

Despite these benefits, however, suprapatellar nailing involves insertion of the trocar into the intraarticular space posterior to the patella [7–9]. This approach places direct pressure on the cartilage and can result in damage via either excess compressive force or abrasive injury upon trocar placement, reaming and nail insertion [7, 10]. Randomized controlled trials and cadaveric studies have both noted the potential for iatrogenic injury to intraarticular structures, such as the menisci, the intermeniscal ligament, the anterior cruciate ligament (ACL), and the patella-femoral joint [1, 11, 12].

The purpose of this report is to present a case of iatrogenic injury to another at-risk structure during suprapatellar tibial nailing: the lateral trochlea cartilage. Additionally, we demonstrate the use of osteochondral allograft for treatment of the cartilage defect.

## CASE REPORT

**Figure 1:**
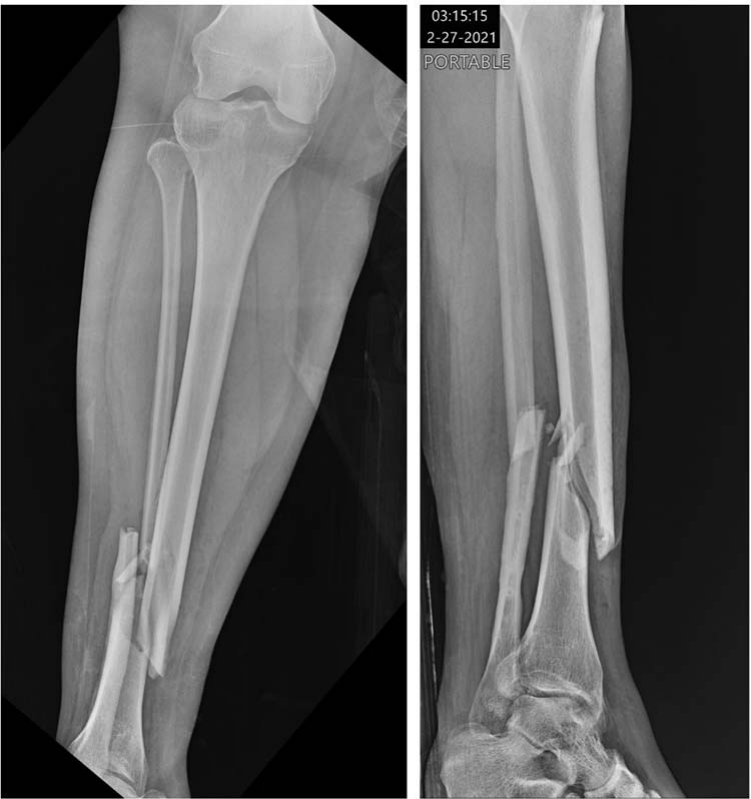
Initial radiographs demonstrating fractures of the proximal fibular neck and distal tibia and fibula.

A 34-year-old obese female (body mass index of 48.17 mg/kg^2^) presented to a level 1 trauma center after experiencing a ground level fall and sustaining a displaced Gustilo-Anderson grade 1 open comminuted spiral fracture of the right distal tibial shaft (OTA classification 42A1c) (Fig. 1). Other injuries included a displaced comminuted distal fibula fracture and a nondisplaced proximal fibular neck fracture. In the emergency department, the 1.5 cm wound over the distal anteromedial tibia was irrigated with 3 L of normal saline, and the fracture was reduced and splinted.

**Figure 2:**
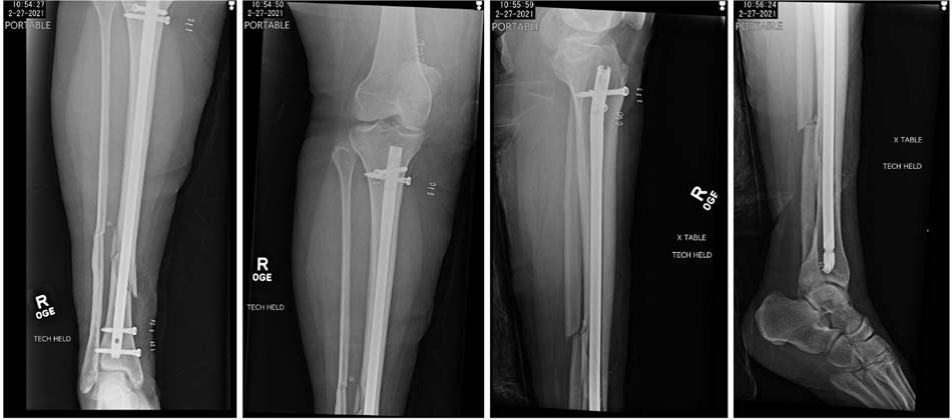
Postoperative radiographs demonstrating reduction of distal tibia/fibula fracture.

The patient was taken to the operating room on the same day for irrigation and debridement and suprapatellar nailing of the tibia. The cannula and trocar wire were driven in place, and the bone was reamed up to 11.5 mm. A 10 mm diameter Smith and Nephew nail was inserted, and two proximal and two distal interlocking screws were placed. No complications nor injury to the cartilage were noted at the time, and imaging demonstrated appropriate reduction of the fracture (Fig. 2). The patient was discharged home the next day with permission to weight bear as tolerated.

**Figure 3:**
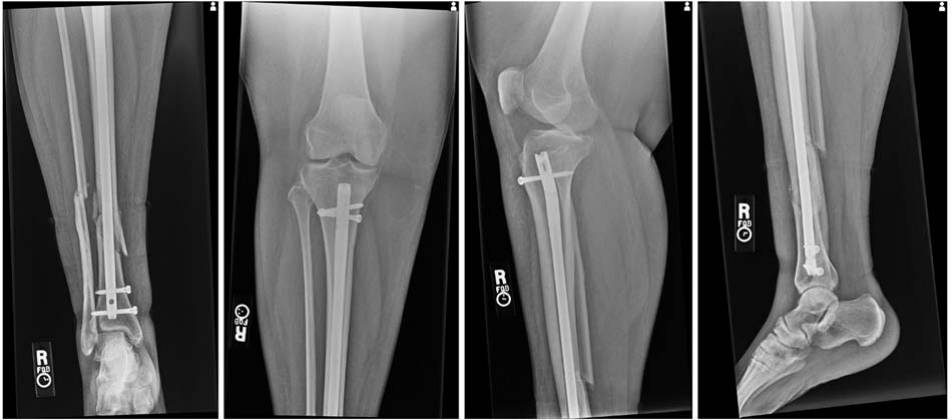
Radiographs at 6 weeks post-op demonstrating unchanged alignment of the distal tibia/fibula fracture.

**Figure 4:**
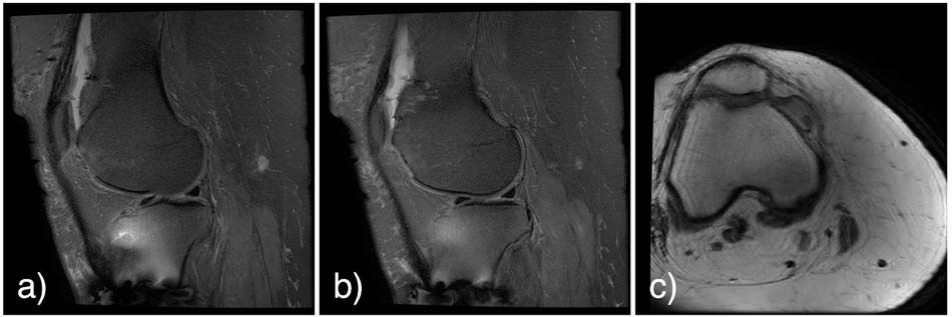
Noncontrast multiplanar T1 (**a** and **b**) and T2 (**c**) MRI at 7 weeks post-op showing a large full-thickness defect on the lateral femoral trochlea measuring 1.8 cm in diameter with a loose chondral fragment in the superior medial joint space.

At 2 weeks post-op, the patient was managing pain well, and she demonstrated appropriate range of motion and strength. At 6-week follow-up, however, she complained of diffuse, painful grinding in the right knee which impaired her ability to ambulate. Physical exam revealed audible and palpable crepitus in the knee as well as tenderness to palpation at the medial and lateral joint lines. Radiographs demonstrated unchanged alignment of the tibia fracture (Fig. 3). Multiplanar T1 and T2 MRI without contrast revealed a large full-thickness defect on the lateral femoral trochlea measuring 1.8 cm in diameter with a loose chondral fragment in the superior medial joint space (Fig. 4).

**Figure 5:**
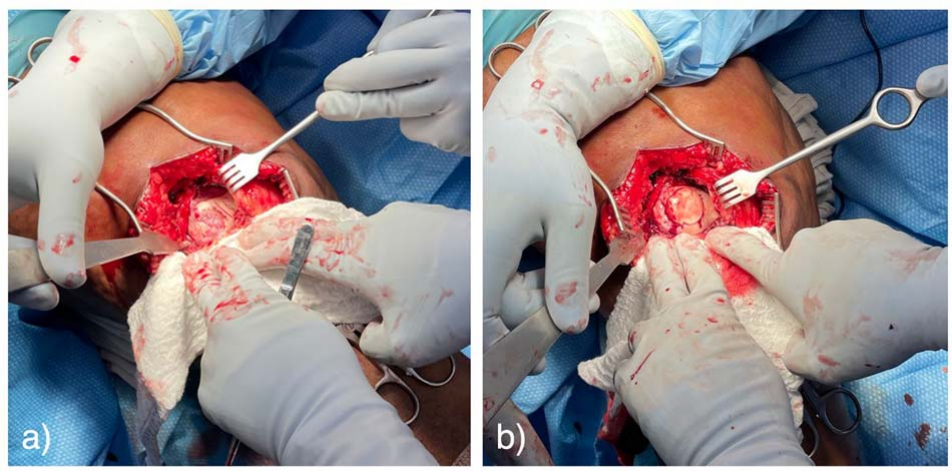
Pictures taken intraoperatively demonstrating full-thickness cartilage defect of the lateral femoral trochlea (**a**), and press-fit osteochondral allograft repair (**b**).

The patient was referred to a fellowship-trained sports medicine orthopedic surgeon. In hopes of relieving the patient’s knee pain, an intraarticular steroid injection was administered. Three weeks after the injection, the patient reported no improvement and continued to be significantly limited by pain. Because the cartilage was deemed unlikely to heal without surgical intervention, she underwent staged reconstruction with a diagnostic arthroscopy and subsequent osteochondral allograft reconstruction of her trochlea cartilage 4 months after her initial injury. In the operating room, the full-thickness chondral defect (Fig. 5a) was cored out, and the loose chondral body was removed. An appropriately sized cadaveric allograft plug was cored out and press-fit into the defect (Fig. 5b).

## DISCUSSION

Intramedullary tibia nailing was first described using an infrapatellar approach [13]. Although a viable option, this technique requires hyperflexion of the knee placing significant strain on the proximal tibia due to increased tension of the quadriceps tendon, relatively frequently resulting in an anterior apex deformity [13]. Instead, nailing via a suprapatellar portal in the semiextended position allows for easier reduction, better facilitates the use of fluoroscopy, and has been shown to result in lower rates of malunion and decreased incidence of anterior knee pain [1–4, 9, 14]. Despite these benefits, however, the suprapatellar technique has also been shown to subject the menisci, intermeniscal ligament, ACL and the patella-femoral joint to iatrogenic injury [11, 12]. To our knowledge, the present cas e is the first to report that the lateral trochlea cartilage is also at risk.

The suprapatellar technique places greater direct pressure on intraarticular cartilage compared to the infrapatellar technique [7]. Contact pressure can be decreased through the use of a trocar system made of elastic synthetic materials, such as thermoplastic polyurethane, or by performing a retinacular release [9]. Iatrogenic cartilage injury is still possible, however, particularly in obese patients, whose excess soft tissues can lead to trocar malposition, and those with decreased patellar laxity and a small joint space, where the trocar can be forced into the cartilage during reaming and nail insertion [15]. To offset the increased pressure on the trocar from a taught patella, the knee can be flex from 15° (semiextended) to 50° in order to accommodate the altered insertion vector [1, 2]. In such cases, however, utilizing the infrapatellar technique may be best to prevent iatrogenic injury [8].

The present case suggests that surgeons must be extremely cognizant of proper guide pin and trocar placement throughout the entire procedure to avoid a potential abrasive injury caused by trocar malposition or slippage. Furthermore, loose trocars may expose the reamer to the intraarticular space and damage cartilage upon bone entry. Therefore, close attention must be paid to the location of the trocar sleeve using fluoroscopy throughout the entire procedure [8].

In conclusion, although a relatively safe procedure, suprapatellar nailing can lead to severe damage to the lateral trochlea cartilage. It is imperative for surgeons to be aware of the potential for iatrogenic injury and to identify the corresponding signs and symptoms in order to allow for early intervention and prevention of further joint damage.
